# Recent advances in intestinal helminth parasites of horses in the Asia-Pacific region: Current trends, challenges and future directions

**DOI:** 10.1016/j.ijpddr.2025.100622

**Published:** 2025-10-17

**Authors:** Ghazanfar Abbas, Martin K. Nielsen, Charles E-Hage, Abdul Ghafar, Ian Beveridge, Jenni Bauquier, Anne Beasley, Edwina J.A. Wilkes, Peter Carrigan, Lucy Cudmore, Caroline Jacobson, Kristopher J. Hughes, Abdul Jabbar

**Affiliations:** a Department of Veterinary Biosciences, Melbourne Veterinary School, The University of Melbourne, Werribee, Victoria, Australia; b Department of Animal and Veterinary Sciences, Aarhus University, Tjele, Denmark; c School of Agriculture and Food Sustainability, The University of Queensland, Gatton, Queensland, Australia; d Racing Victoria, Flemington, Victoria, 3031, Australia; e Scone Equine Hospital, Scone, New South Wales, 2337, Australia; f Centre for Animal Production and Health, Murdoch University, Murdoch, Western Australia, 6150, Australia; g School of Agricultural, Environmental and Veterinary Sciences, Charles Sturt University, Wagga Wagga, New South Wales, Australia

**Keywords:** Ascarids, Strongylids, Horse, Australia, New Zealand, Anthelmintic resistance, Parasite control guidelines

## Abstract

Over the past 25 years, significant progress has been made in understanding and managing equine gastrointestinal parasites in the Asia-Pacific region, particularly in Australia and New Zealand. This review synthesises current knowledge of the epidemiology, diagnostic methods, anthelmintic resistance (AR), and control strategies for major equine intestinal parasites, including cyathostomins, *Parascaris* spp., *Anoplocephala perfoliata*, and *Strongyloides westeri*. Recent studies highlight substantial regional variation in parasite prevalence, egg shedding and cyathostomin population composition, shaped by diverse climatic conditions. Of increasing concern is the emergence of resistance to commonly used anthelmintics which is now evident in both *Parascaris* and cyathostomins, although data for *S. westeri* and *A. perfoliata* remain limited. High-throughput molecular diagnostics, such as next-generation sequencing, have advanced species-level characterisation in Australia and Thailand. ELISA-based tests for *A. perfoliata* and encysted cyathostomins are promising but remain unvalidated and underutilised regionally. The routine use of combination anthelmintics, including benzimidazoles, praziquantel, pyrimidines, and macrocyclic lactones, may accelerate resistance across nematode and cestode populations, emphasising the need for regular efficacy monitoring and improved antiparasitic stewardship. Findings from recent research on horse parasites in Australia have informed the development of country's first national equine parasite control guidelines which recommend targeted or selective treatment strategies. However, the effectiveness of these strategies requires ongoing evaluation, particularly in year-round grazing systems in tropical and subtropical regions. Sustainable parasite control will depend on the integration of non-chemical strategies along with the use of anthelmintics and the establishment of a national parasite surveillance database. This review highlights the need for climate-specific treatment protocols, strengthened collaborative research infrastructure, and continued investment in innovative diagnostic and control methods to preserve equine health and anthelmintic efficacy across the region.

## Highlights

• The review provides a comprehensive overview of equine intestinal helminths in the Asia-Pacific region.
• Australia's first national equine parasite control guidelines promote targeted selective treatments.
• While research has advanced on cyathostomins and *Parascaris* species, *Anoplocephala perfoliata* remains unexplored.
• Surveillance, integrated farm-specific strategies are key to parasites and drug resistance control.
• Studies on the impact of climate on parasite transmission dynamics are urgently needed.

## Graphical abstract

**Figure:**
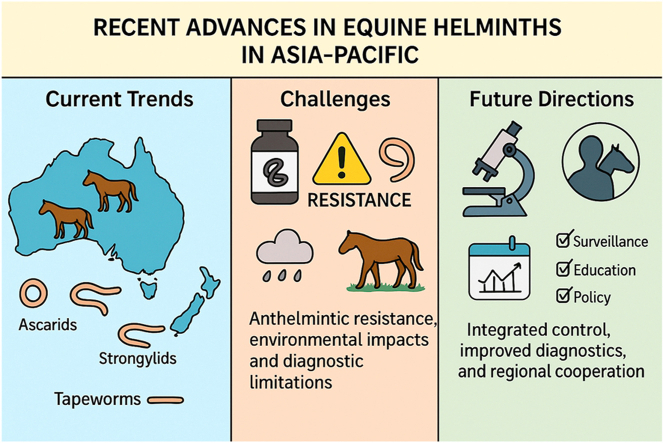

## 1 Introduction

Horses around the world are exposed to a variety of helminth parasites, and while many of them are considered ubiquitous, regional differences require specific considerations. Over the past two decades, substantial research from Australia and New Zealand has advanced understanding of the epidemiology, diagnosis, control and resistance levels to commonly used anthelmintics for gastrointestinal nematodes in horses. This research has been pivotal in recognising the emergence and spread of anthelmintic resistance (AR) among key nematode species (i.e., cyathostomins and *Parascaris* spp.), which has urged a paradigm shift from traditional interval-based deworming to evidence-based control strategies in the region. While international guidelines for equine parasite control exist (Nielsen et al., 2025a), parasite burdens and control practices differ across the region, mainly due to distinct climate, seasons and available anthelmintic options (commonly available combination products), highlighting the need for tailored regional approaches for parasite control in the southern hemisphere. In response, a recently completed national equine parasitology research project in Australia resulted in a peer-reviewed parasite control guideline document (Beasley et al., 2025) and presented an opportunity to discuss and compare recommendations to those formulated in other guideline documents published for different countries around the world (Nielsen et al., 2025a). While this exercise generated useful information and pointed to several possible differences between Australia and Northern Hemisphere countries, it also demonstrated a need for seeking a deeper investigation into recent equine parasitology research advances in the Eastern/Southeastern Asian and Southern Pacific regions to better understand pertinent questions and challenges within this region and identify research needs going forward.

Thus, this review provides insights into the research on helminth parasites of horses conducted in the past 25 years in the Asia-Pacific region. It also identifies region-specific challenges and highlights knowledge gaps to inform future research and policy development.

## 2 Intestinal parasite species

Ascarid and strongylid nematodes are generally considered to be ubiquitous targets for parasite control and they are also clearly demonstratable in this region. Although other parasites have been investigated in various studies, most research has focused on ascarid and strongylid nematodes. The following sections provide a summary of research conducted on important parasites of horses in the Asia-Pacific region.

### 2.1 Ascarids

*Parascaris* spp. are the largest roundworms of horses and are highly prevalent in foals and juvenile horses. The parasite primarily resides in the small intestines of young horses up to 2 years of age but can occasionally be found in adult or geriatric horses (Abbas et al., 2023a, 2024a). In foals and weanlings, heavy worm burdens can lead to poor growth, colic and intestinal obstruction (Cribb et al., 2006). Horses develop protective, strong immunity against ascarids by one year of age, thereby significantly reducing the occurrence of patent infection in horses beyond two years of age (Reinemeyer, 2012). Earlier studies in Australia reported 58 % of the foals shedding ascarid eggs in their faeces (Armstrong et al., 2014). Recently, two studies reported ascarid egg shedding prevalences in foals (35 % and 46 %), weanlings (21 % and 32 %), yearlings (10 % and 13 %) and adults (<1 %) in Australia (Abbas et al., 2023a, 2024a). Similarly, another Australian study reported an individual ascarid prevalence in foals of 27 % and a farm prevalence of 33 % (Beasley et al., 2020). A study conducted in New Zealand determined monthly faecal egg counts (FECs) in two cohorts of foals and found the ascarid prevalence peaked at 4–5 months of age, reaching approximately 50 % in the lesser treated group (Nielsen et al., 2021), after which the prevalence declined substantially at 6 and 7 months of age. Similarly, a recent study from Thailand reported 3.8 % prevalence of ascarid eggs in foals’ samples collected from 11 farms (Phetkarl et al., 2024).

The existence of two cryptic *Parascaris* species – *P. univalens* and *P. equorum* – has attracted increasing attention in recent years (Nielsen et al., 2014; Jabbar et al., 2014). The two species are morphologically alike and can only be differentiated by karyotyping (Goday and Pimpinelli, 1986). Karyotyping studies performed in recent years have only identified *P. univalens* (Nielsen et al., 2014; Martin et al., 2021a, 2021b; Samson-Himmelstjerna et al., 2021) and the last confirmed identification *of P. equorum* was published in 1986 (Goday and Pimpinelli, 1986). In the last decade, Chinese researchers have contributed substantially to understanding the population genetics of *Parascaris* spp. (Gao et al., 2019; Peng et al., 2019a). Recently, a Chinese study reported the existence of an apparent third *Parascaris* species characterised by having six chromosomes as opposed to the two and four chromosomes found in *P. univalens* and *P. equorum*, respectively (Zhou et al., 2023). Interestingly, a trivalent (three chromosome pairs) equine ascarid had been described in China in the 1930s (Li, 1937), but not since then. Thus, more work is required to explore the existence of different *Parascaris* species in various equid populations in Asia-Pacific and around the world.

### 2.2 Strongylids

While strongylids are generally considered ubiquitous in grazing horses, large strongyles, in particular *Strongylus vulgaris*, receive attention due to their well-described pathological potential (Nielsen et al., 2016; Pihl et al., 2018; Walshe et al., 2021). An Australian survey reported *S. vulgaris* prevalences of 6.9 % and 7.8 % determined by coproculture and PCR, respectively (Beasley et al., 2020), which is similar to recent findings reported from Scandinavia (Tydén et al., 2019; Nielsen et al., 2025b), where the parasite is considered enzootic. However, two Australian studies using the metabarcoding detection method found *Strongylus* spp. at substantially lower levels. One study found a low occurrence of *S. equinus* (0.83 %) and only in mares, but did not detect *S. vulgaris* (Abbas et al., 2024a). A different study found evidence of all three *Strongylus* species; *S. vulgaris* (0.2 %), *S. edentatus* (1.4 %), and *S. equinus* (0.01 %), but at very low prevalences (Abbas et al., 2023a). Similarly, metabarcoding studies carried out in Thailand found no evidence of *Strongylus* spp. (Hamad et al., 2024a, 2024b). In contrast, another study in Thailand found *S. vulgaris* in all pooled samples from 11 farms using PCR (Phetkarl et al., 2024). Reduced dominance of *Strongylus* spp. is due to reliance on routine and intensive macrocyclic lactone (ML) treatment in the region. Early studies from New Zealand and Australia found a visible shift in prevalence from *Strongylus* spp. to cyathostomins (Dargatz et al., 2000; Leathwick et al., 2001). Later, researchers from both countries reinforced that *Strongylus* spp. had significantly declined to near absence in horses (Flanagan et al., 2013, Sutherland and Leathwick, 2011). However, an Australian study on feral horse populations demonstrated that >96.7 % of horses harboured *S. vulgaris* (Harvey et al., 2019), constituting a reservoir for the parasite and a potential threat to domestic horses. Thus, large strongyles appear to deserve attention in Australian horse populations and should be considered in parasite control programs.

The species composition of cyathostomin parasite burdens is a topic that is gaining attention due to the recent advent of metabarcoding (“nemabiome”) diagnostic techniques. A meta-analysis of equine necropsy studies performed primarily in Europe and North America over five decades has demonstrated that the three most abundant species in naturally infected horses are *Cylicocyclus* (*Cyc*.) *nassatus*, *Cylicostephanus* (*Cys*.) *longibursatus*, and *Cyathostomum* (*Cya*.) *catinatum*, which together comprise about 55 % of recovered specimens (Bellaw and Nielsen, 2020). The predominance of these three species has been generally confirmed by recent metabarcoding studies conducted in the United Kingdom (Bull et al., 2024), France (Boisseau et al., 2023), and the US (Nielsen et al., 2022). In this context, it is interesting to note that metabarcoding studies from Australia and Thailand revealed some notable differences. For example, *Cya. catinatum* was generally rare (about 2 % or less) in both countries, whereas *Cys. longibursatus* was consistently highly abundant (Abbas et al., 2023a, 2024a; Hamad et al., 2024a, 2024b). The third species considered to be globally highly abundant is *Cyc. nassatus*, which was found in high abundance in some populations, but not in others (Abbas et al., 2023a, 2024a; Hamad et al., 2024a, 2024b). Thus, some differences appear between regions, potentially reflecting climatic conditions, treatment regimens, or other unidentified factors. However, it should be noted that *Cys. longibursatus* and *Cyc. nassatus* were associated with shortened strongylid egg reappearance periods following ivermectin (IVM) administration in Thailand (Hamad et al., 2024c). Similarly, *Cyc. nassatus* was reported to be the dominating species following IVM and moxidectin (MOX) administration in Australia, and, interestingly, *Cya. catinatum* was abundant following abamectin (ABM) administration (Abbas et al., 2024b). Thus, it appears that these three species might be associated with ML resistance, and it is possible that relative abundance could increase with increasing resistance levels.

### 2.3 Strongyloides westeri

*Strongyloides westeri*, commonly known as threadworm, is typically the first parasite to establish patent infections in foals, primarily infecting individuals up to 16 weeks of age. A recent study from Australia reported the presence of *S. westeri* eggs in a foal at a thoroughbred stud farm (Abbas et al., 2021b). Similarly, *S. westeri* eggs were found in a 20-week-old foal in Thailand (Phetkarl et al., 2024). In New Zealand, *S. westeri* was detected in seven and 22 foals in pre-treatment samples collected for two FEC reduction tests (Morris et al., 2019). To date, there are no confirmed reports of anthelmintic resistance in this species. In the aforementioned treatment trials, only one foal continued to shed eggs following treatment with IVM (Morris et al., 2019). However, this finding does not necessarily indicate resistance, as *S. westeri* egg shedding is intermittent. Continued monitoring remains important, especially considering that only a limited number of anthelmintics (such as IVM, oxfendazole (OFZ) and fenbendazole (FBZ)) are available for treatment.

### 2.4 Setaria spp

*Setaria digitata* deserves mention due to its presence in the region and the severe clinical implications of infection. This is a filarial nematode parasite, which is transmitted by mosquitoes and is described to primarily infect cattle (Mohanty et al., 2000). However, multiple reports from the Southeast Asian region demonstrate that the parasite is capable of infecting horses, causing severe ocular disease, including blindness (Shin et al., 2017) or neurological manifestations such as hindlimb ataxia (Lee et al., 2021). In recent years, such cases have been reported from South Korea (Shin et al., 2017; Hwang et al., 2022), Malaysia (Peng et al., 2019b), Thailand (Junsiri et al., 2023), and China (Yu et al., 2021). Thus, *S. digitata* is a severe equine pathogen in this region with potentially fatal consequences.

### 2.5 Cestodes

Globally*, A*. *perfoliata* is considered the primary equine cestode parasite (Nielsen, 2016), but relatively scant information is available from the Southeast Asian and Pacific regions. An Australian survey reported a horse-level prevalence of 0.5 % based on modified McMaster FECs and a farm-level prevalence of 3.9 % based on PCR (Beasley et al., 2020). This is similar to observations reported from other parts of the world, where a very small percentage of faecal samples have been positive for tapeworm eggs (Jürgenschellert et al., 2020). While PCR is potentially more sensitive for parasite detection (Drogemüller et al., 2005), current evidence suggests that this method might not offer substantial advancements compared to standard FEC techniques (Traversa et al., 2008). Thus, the true prevalence of *A. perfoliata* in Australia could be higher than reported, but this is currently unknown, and the occurrence and clinical implications of this parasite should be a research priority in this region.

Outside of Australia, very few studies have reported aspects of *A. perfoliata* infection in the region. The only recent exceptions include a collaboration between researchers in Malaysia and the UK evaluating the *in vitro* efficacy of papaya-derived cysteine proteases against *A. perfoliata* (Mansur et al., 2016) and a UK/Thailand collaboration reporting on the *A. perfoliata* secretome (Wititkornkul et al., 2021; Northcote et al., 2024). One Japanese study reported an increase of *A. perfoliata* eggs in faecal samples following administration of a bithionol anthelmintic (Sanada et al., 2009). Taken together, very little is known about this parasite in the Southeast Asian and Pacific regions, and it should be given more attention in future research projects.

## 3 Epidemiology and the effect of climatic differences

Climate significantly influences the transmission and persistence of parasitic diseases, particularly those involving pasture-contaminating stages such as the eggs and free-living third-stage larvae (L3) of nematodes. Warm and moist conditions in temperate regions accelerate larval development and hatching, while hot and dry environments tend to reduce parasite survival and infection rates. Researchers from New Zealand modelled climate data (temperature and rainfall), cyathostomins free living stages and development of resistance to check the influence of climate on level of parasitism, survival of infectious stages in the environment and development of AR in early, mid and end of 21st century, and found that climate change will significantly influence both level of parasitism (various environmental conditions such as winters with less cold days seemed suitable for longer larval survival) and AR development (Leathwick et al., 2019; Sauermann et al., 2019, 2020). Due to changing environmental conditions, the authors also suggested that sustainable control of parasites will no longer be sufficiently achieved through anthelmintic treatments alone in certain climates (e.g., temperate) (Sauermann et al., 2020). However, the scale of climate impact will vary with the magnitude of climatic changes (i.e., temperature, humidity and rainfall patterns) and among climatically different locations (Sauermann et al., 2020). Australia's diverse climate, characterised by variable rainfall and temperature patterns, plays a critical role in shaping parasite epidemiology. According to the Bureau of Meteorology (BOM), the continent is divided into six climatic zones based on temperature and humidity, ranging from ‘hot humid summers’ in the north to ‘mild/warm summers, cold winters’ in the south and southeast. Similarly, rainfall-based classifications, used by BOM and WormBoss (for the control of sheep parasitic nematodes), could be classified into four major rainfall zones: summer-dominant, winter-dominant, non-seasonal and Mediterranean. Recent Australian research has highlighted significant differences in parasite burdens across these zones, with higher parasite egg burdens observed in Mediterranean regions (Abbas et al., 2023a, 2024a). Moreover, seasonal weather patterns, particularly in autumn, have been associated with increased faecal egg shedding in horses, further underscoring the importance of climate in shaping parasite epidemiology.

## 4 Diagnostic methods

A recent systematic review summarised the large body of literature evaluating FEC techniques for equine parasites and pointed out knowledge gaps and research needs (Ghafar et al., 2021). This review reported a lack of consensus on the egg counting methodologies/protocols and the performance measures used to evaluate and validate these methods. In recent years, several artificial intelligence-based egg counting methods have been developed, and several have been made commercially available (Slusarewicz et al., 2016; Elghryani et al., 2020; Nagamori et al., 2020; McEvoy et al., 2024). An image analysis-based egg counting device has been produced in New Zealand and validated for equine usage (Tyson et al., 2020) and more such devices can be expected in the future. It is hoped that such semi-automated diagnostic methods will facilitate an increase in the surveillance of ascarid and strongylid nematodes in horses as well as the monitoring of the efficacy of anthelmintics against these parasites.

Various molecular techniques such as PCR, restriction fragment length polymorphism, nested-PCR, PCR-directed next-generation sequencing, Southern blotting, single strand conformation polymorphism, PCR-enzyme linked immunosorbent assay, matrix-assisted laser desorption/ionisation-time of flight and random amplification of polymorphic DNA have mostly targeted the second internal transcribed spacer (ITS-2) of the nuclear DNA for molecular detection and characterisation of strongylids (Ghafar et al., 2023). The metabarcoding technique for detecting and semi-quantifying cyathostomin species has been successfully employed in studies conducted in Thailand (Hamad et al., 2024a, 2024b) and Australia (Abbas et al., 2023a, 2024a). This technique will be helpful for future studies to understand cyathostomin species composition dynamics under various climatic conditions, in different breeds and age groups, in relation to parasitic disease, and drug resistance profiles.

Recently, Abbas et al. (2021b) reported successful PCR detection of *S. westeri* DNA in the faeces from a foal harbouring a patent infection of this parasite. This creates the opportunity to conduct large-scale epidemiological and phylogenetic studies of this parasite. Similarly, a single study employed a PCR targeting ITS-2 to detect *A. perfoliata* in Australia and revealed 3.9 % (of 102) properties positive for the parasite (Beasley et al., 2020). Various other diagnostic methods have been developed for the detection of equine tapeworms due to the low sensitivity of FEC methods (usually between 8 and 62 %) and difficulty in identifying burdens less than 20 tapeworms (Lightbody et al., 2016; Proudman and Edwards, 1992). These methods include ELISA tests based on antibody detection in serum and saliva samples (Lightbody et al., 2016), and offer valuable herd-level insights into tapeworm exposure. However, the reliability of these methods for individual diagnosis remains limited (Nielsen, 2016). Given the reliance on only two anthelmintic compounds (i.e., praziquantel (PZQ) and pyrantel (PYR)) for treating equine tapeworm infections, and their widespread inclusion in combination dewormers available in Australia (Abbas et al., 2023b, 2024c; Wilkes et al., 2017), routine surveillance of tapeworm prevalence and anthelmintic efficacy using appropriate and validated diagnostic tools is lacking and strongly recommended.

## 5 Genomic studies

In recent years, substantial progress has been made in equine parasite genomic studies. Mitochondrial genomes have been sequenced and annotated for a large number of equine helminth parasites, and scientists in China have conducted much of this work. Below is a summary of this work.

Complete mitochondrial genomes have been published for several species within the subfamily Strongylinae, including *Triodontophorus brevicauda, T. nipponicus,* and *T. serratus* (Duan et al., 2015, 2017; Gao et al., 2017a). Phylogenetic analysis, based on genomic and mitochondrial DNA markers, has suggested that the *Triodontophorus* genus should belong to the Cyathostominae (Gao et al., 2017b; Hung et al., 2000). Furthermore, the mitochondrial genome of *S. equinus* has been published (Xu et al., 2015).

Among the Cyathostominae, mitochondrial genomes have been published for several species, including *Cyc. nassatus* (Gao et al., 2017c), *Cyc. radiatus* (Hu et al., 2020), *Cya*. *pateratum* (Qiu et al., 2018), *Cya. catinatum* (Qiu et al., 2018), *Cys*. *longibursatus* (Ma et al., 2024), *Coronocyclus labiatus* (Gao et al., 2021), and *Cylicodontophorus bicoronatus* (Gao et al., 2021). In addition, a comparative analysis of mitochondrial DNA data from four *Cys. minutus* isolates suggested that this species may represent a species complex, indicating the presence of cryptic species (Gao et al., 2020). It was first indicated using genomic DNA markers (ITS-2) in the early 2000s (Hung et al., 1999) and has recently been confirmed in a study that used the cytochrome *C* oxidase subunit I gene for cyathostomin metabarcoding and found evidence of three species belonging to the *Cys. minutus* complex (Diekmann et al., 2025). The same study also demonstrated that cryptic species exist within the *Cys. calicatus* complex (Diekmann et al., 2025), and it is clear that more work is needed on the complexity and species diversity of the cyathostomin subfamily using molecular markers other than ITS-2.

Apart from the Strongylida, mitochondrial genomes have been published for *Anoplocephala magna* (Guo, 2016), *Setaria digitata* (Liu et al., 2017) and *Parascaris* spp. (Gao et al., 2019). In addition, a whole genome sequencing study of *P. univalens* from different equid hosts has provided novel and useful insights into the evolution and gene flow within this species (Han et al., 2022).

## 6 Anthelmintics

While the same four anthelmintic classes (benzimidazoles (BZs), pyrimidines, MLs, and isoquinoline-pyrazines) are generally available worldwide, there are some regional differences in the range of anthelmintic products available for use in horses. For example, combination products with three or four different anthelmintic classes exist in New Zealand and Australia (Scott et al., 2015; Abbas et al., 2023b), whereas the only combinations typically available elsewhere are products containing PZQ and an ML. In Australia, products combining pyrimidine-class drugs (morantel [MOR] or PYR) with other drug classes are available. Additionally, ABM, an ML, is marketed in both New Zealand and Australia, in single as well as combination formulations (Scott et al., 2015; Abbas et al., 2023b). Neither of these is widely available for equine use elsewhere in the world.

Combining different anthelmintic pharmaceutical classes into a single product is theoretically justified by two aims: (i) ensuring high treatment efficacy, and (ii) reducing the development of AR. These benefits are supported by computer simulation studies (Sauermann et al., 2019; Leathwick et al., 2019; Scare et al., 2020). However, it should be emphasised that these simulated scenarios are based on combining novel anthelmintics to which no resistance has yet developed. This contrasts starkly with the real-life situation, where AR has been reported in multiple equine parasites to all currently available classes (Nielsen, 2022). While some simulations suggest potential benefits of including anthelmintics with reduced efficacy in combination products (Scare et al., 2018), there is no information about the long-term efficacy and sustainability of combining several anthelmintic classes to which high levels of AR already exist. Thus, the anthelmintic performance of all combination anthelmintic products should be monitored closely in the years to come.

### *6.1* Resistance in ascarids

Several recent studies have documented reduced single active anthelmintic efficacy against ascarid and strongylid nematodes in the region. Studies in New Zealand and Australia have provided evidence of resistance to IVM, ABM, and MOX in ascarids (Armstrong et al., 2014; Beasley et al., 2015; Morris et al., 2019; Abbas et al., 2024b). Resistance to all available single active MLs, such as ABM, IVM and MOX has been reported in ascarids in Australia (Abbas et al., 2024b; Wilkes et al., 2017). Additionally, resistance in *Parascaris* has also been found to FBZ, PYR (Armstrong et al., 2014), OFZ and its combination with PYR (Abbas et al., 2024b), the drugs generally regarded to be the most effective against ascarids. Although MOR and PYR salts are not available as single active formulations in Australia, Armstrong et al. (2014) imported PYR for their study and found resistance in *Parascaris* to the drug (Armstrong et al., 2014).

Recent work has provided information about the anthelmintic efficacy of several combination products in New Zealand and Australia. When the ML-resistant ascarids were treated with a combination of ABM-MOR, this combination had a FECR of >99 % demonstrating that the combination still effectively controls ML-resistant worms (Wilkes et al., 2017). Similarly, a combination of OFZ and PYR has been documented to have >95 % efficacy against ascarids (Morris et al., 2019; Abbas et al., 2024b), but <90 % efficacy against strongylids on a majority of farms (Abbas et al., 2021a, 2024b).

Taken together, these data suggest that this combination might have value against ascarids but is unlikely to be fully effective against strongylids. As a general finding, combinations including an ML (IVM or ABM) displayed high efficacy against strongylids (Morris et al., 2019; Abbas et al., 2021a, 2024b), but a limited number of products have been evaluated at this stage, and more data are needed on the performance of combination products.

### 6.2 Resistance in strongylids

Several recent studies have documented single active anthelmintic efficacy against strongylid nematodes in the region. Studies in New Zealand and Australia have provided evidence of resistance to IVM, ABM and MOX in strongylids (Abbas et al., 2021a, 2024b; Scott et al., 2025). Resistance in strongylids has also been documented to the most commonly used combination product (OFZ and PYR) in Australia (Abbas et al., 2024b). Furthermore, evidence of shortened strongylid egg reappearance periods (ERPs) by four weeks following ML treatments has been reported from Australia (Abbas et al., 2021a, 2024b), New Zealand (Rosanowski et al., 2017; Scott et al., 2025) and Thailand (Hamad et al., 2024c). These findings are aligned with general trends of reduced ML efficacy reported worldwide (Macdonald et al., 2023; Nielsen, 2022). The interpretation of shortened ERP has been debated in recent years. It was once considered an indicator of emerging anthelmintic resistance (Sangster, 1999), but now also thought to reflect selection for biological traits such as species or isolates with shorter life cycles (Nielsen et al., 2022, 2023) Interestingly, Scott et al. (2025) suggested that ERP estimates may be affected by season, demonstrating that more work is needed to fully understand the use of ERP as an anthelmintic performance metric.

In addition, extensive resistance to BZ compounds has been demonstrated in strongylid parasites (Pook et al., 2002; Morris et al., 2019; Abbas et al., 2024b). This was also evident in a study from the early 2000s, where Pook et al. (2002) reported treatment failures with oxibendazole and MOR against strongyles on six and two properties, respectively (Pook et al., 2002). However, limited data are available for their efficacy in the region due to the unavailability of pyrimidines as single actives (primarily marketed as combination products with BZ and ML).

## 7 Alternatives to anthelmintic medication

A few studies have evaluated potential novel modalities for equine parasite control, including the *in vitro* study of papaya extract activity against *A. perfoliata* (Mansur et al., 2016). Recently, an important development occurred where a product containing the spores of a nematophagous fungus, *Duddingtonia flagrans* was commercialised (Healey et al., 2018). This product is based on feeding fungal spores to grazing animals, including horses, and upon being passed in the faeces, the spores germinate and form a network of hyphae, trapping and killing nematode larvae. Plot studies have demonstrated that feeding this product to grazing horses can significantly reduce the number of strongylid larvae within faecal pats (Healey et al., 2018). However, several questions remain about how the product can be integrated into an equine parasite control program in conjunction with anthelmintic treatments and other management practices and an Australian questionnaire survey found that only about 9 % of veterinarians were incorporating the use of *Duddingtonia flagrans* spores in their parasite control program recommendations (Abbas et al., 2023b, 2024c).

Earlier work in Australia has demonstrated the potential for using dung beetles for equine parasite control (English, 1979; Mfitilodze and Hutchinson, 1988), but this has not been investigated recently. The idea is that the beetles facilitate the disintegration of the faecal pats, reducing the survival of strongylid larvae primarily through desiccation. However, a recent review summarising studies on the utility of dung beetles for parasite control in cattle concluded that the evidence supporting this concept is minimal and may have been overestimated in the past (Forbes and Scholtz, 2024).

A recent questionnaire survey conducted among Australian thoroughbred managers demonstrated that some forms of pasture management were regularly employed as part of an integrated parasite control program (Abbas et al., 2024c). These included pasture hygiene, co- or alternate grazing with ruminants, harrowing/mowing and rotational grazing. Thus, it is apparent that this study population is well-informed about the value of such non-chemical means for parasite control in horses. However, the majority of respondents in this study continued to use interval-based deworming across all age groups of horses, which may undermine the effectiveness of non-chemical control measures. Similarly, respondents from New Zealand reported a continued reliance on interval treatments and limited on-farm parasite control practices on breeding and training farms (Bolwell et al., 2015; Rosanowski et al., 2016). Given these trends, it may be worthwhile re-visiting research on how effective these management methods are for reducing pasture contamination with current worm species, pasture management practices and changing weather patterns across the region.

## 8 Discussion, conclusions and future implications

In the last 25 years, substantial progress has been made within various aspects of equine parasitology research across the Asia-Pacific region. This includes novel insights into cyathostomin species composition in various cohorts of horses, both before and following anthelmintic administration, as well as the data on the efficacy of anthelmintic treatments against ascarid and strongylid nematodes. Despite this substantial progress in understanding equine parasitology, particularly in Australia, several critical questions and research needs remain. Given the global challenges associated with AR, continued monitoring of parasites af AR remain critical going forward. This review has also identified several research needs and knowledge gaps, including a lack of assessment of the risks of parasitic disease for horses infected with these parasites, very limited information about the prevalence and importance of *A. perfoliata* in the region, little or no use of advanced serological or molecular tools for parasite detection and AR monitoring, a paucity of comprehensive studies on pasture egg burdens and larval contamination, and limited understanding of the long-term implications of using combination anthelmintic products.

Although Australia ranks second only to the United States in terms of Thoroughbred horse population and features diverse climates and distinct seasons, research on equine parasites remains limited compared to countries like the USA and the UK. Table 1 outlines key future research priorities in equine parasitology in the Asia-Pacific region. More research into the impact of parasites on equine health, welfare and disease management is among the more prominent needs. Except for the herein summarised case studies of equine *S. digitata* infection (Shin et al., 2017; Peng et al., 2019b; Yu et al., 2021; Hwang et al., 2022; Junsiri et al., 2023) and one New Zealand study evaluating measures of health in cohorts of horses (Nielsen et al., 2021), very little emphasis has been placed on health aspects of equine parasitism. This situation is not exclusive to this region but represents a global trend. Currently, very little is known about the risks of parasitic disease and how to mitigate these in managed horses globally. However, current guidelines, including recently published Australian equine parasite control guidelines (Beasley et al., 2025) aim to minimise the risk of parasitic disease. Thus, a better understanding of disease risks and health implications of equine parasite infection is strongly warranted and should be made a top research priority to avoid indiscriminate use of anthelmintics. Furthermore, targeted research into the relationship between parasitic burden and athletic performance in racehorses could offer valuable insights for the health and welfare of horses.

**Table 1:** Future research needs in equine parasitology within the Asia-Pacific region.

1 Identify the specific *Parascaris* species currently prevalent in the field and determine which are associated with AR
2 Conduct dedicated research to understand the impact of climate on parasite distribution and infection intensity in both pastures and horses
3 Investigate the prevalence and burden of *A. perfoliata* through targeted epidemiological studies using serological, molecular and faecal flotation methods
4 Assess industry uptake and implementation of newly developed Australian guidelines on parasite control practices, identifying barriers to adoption and areas needing further extension or clarification
5 Undertake longitudinal studies to demonstrate the use of integrated parasite management strategies (i.e., chemical and non-chemical) in horses and manage AR

While the majority of the work in the region has focused on ascarid and strongylid nematodes, and understandably so, it is remarkable that very little attention has been given to *A. perfoliata*. This cestode is recognised as a major equine pathogen worldwide and is considered ubiquitous in grazing horses (Nielsen, 2016). Furthermore, recent reports of suspected anticestodal resistance in the US (Nielsen, 2023; Finnerty et al., 2024) have further emphasised the need for more research on this parasite. In Australia and New Zealand, nearly all commercial equine dewormers combine two or more active ingredients, with the most commonly available formulation of ML and BZ combined with PYR and/or PZQ (Abbas et al., 2023b, 2024c; Wilkes et al., 2020). The routine administration of PYR and/or PZQ, regardless of whether cestode infection is present, raises significant concerns regarding sustainability and the potential acceleration of AR due to unnecessary selection pressure. In effect, horses are often exposed to these compounds multiple times a year (Abbas et al., 2024c; Wilkes et al., 2020), which not only risks the emergence of resistance in *A. perfoliata*, but also undermines the principle of targeted treatment central to sustainable parasite control guidelines (Beasley et al., 2025). Thus, the prevalence, clinical importance of *A. perfoliata* and efficacy of available dewormers need to be studied in the region, and possible parasite control measures should be considered.

The widespread use of combination anthelmintic products in Australasia represents a unique scenario substantially distinct from elsewhere. While the work referenced herein has demonstrated that combinations can be effective treatment options against ascarid and strongylid nematodes, the data illustrated that resistance can develop to combination products (Morris et al., 2019; Abbas et al., 2021a, 2024b). Furthermore, the long-term consequences of using such products are unknown. Will these combination products slow down resistance development or increase it? Will these products promote multidrug resistance? These questions remain unanswered, and it is, therefore, of utmost importance to monitor the efficacy of these products against both ascarid and strongylid nematodes. In this context, equine veterinarians can play a crucial role in implementing routine efficacy testing on farms as recommended in the Australian parasite control guidelines (Beasley et al., 2025). Thus, clear and effective communication of these practices to horse owners/breeders/farm managers by veterinarians, along with practical testing guidelines, will be critical for sustainable implementation.

Beyond the implication for AR management, the routine and widespread use of anthelmintics, particularly those excreted largely unchanged (e.g., IVM) or active metabolites, raises serious concerns about environmental impact. These compounds can enter the environment through direct excretion, posing significant toxic effects to insects, such as dung beetles, earthworms and aquatic animals or indirectly to the food chain (Haseler et al., 2024). While specific data in equines are limited, a growing body of research in ruminants and other livestock highlights these risks (Beynon, 2012), warranting similar attention in the equine sector. In this context, the implementation of targeted selective treatment (TST) strategies offers a sustainable alternative. By reducing the frequency of anthelmintic administration, TST not only helps preserve drug efficacy and delay AR development in an era of limited treatment options availability and shrinking pipeline of new development for equine parasites but also helps to mitigate environmental contamination.

Along with this, limited treatment options for foals might increase the prevalence of other important parasites, such as *S. westeri*, especially when anthelmintic products are deemed unsuitable against *Parascaris* and strongyles due to resistance concerns. With only three drugs (IVM, OFZ, and FBZ) known to be effective against *S. westeri*, managing concurrent infections in young horses could become increasingly challenging. Moreover, the epidemiology of *S. westeri* in the Asia–Pacific region remains poorly understood. In contrast, the parasite has been well studied in the United States. Historically, *S. westeri* was highly prevalent among foals in central Kentucky and other regions during the early 20th century. However, from the mid-20th century to the early 2000s, prevalence declined markedly due to the widespread use of effective anthelmintics, including BZs and MLs, often administered primarily for the control of ascarid and strongylid nematodes (Lyons and Tolliver, 2014a; Lyons et al., 1993). Interestingly, a resurgence in the prevalence was observed in central Kentucky, where *S. westeri* infection rates in foals rose from <6 % (in the late 1900s and early 2000s) to approximately 30 % in 2014 (Lyons and Tolliver, 2014b). This increase was ascribed to a reduced IVM use in foals, stemming from concerns over emerging resistance in *Parascaris* spp. (Lyons and Tolliver, 2014a).

Climatic considerations are highly relevant in parasitology due to the implications for parasite transmission and the possible changes in these dynamics caused by climate change (Sauermann et al., 2020). By collecting data across diverse Australian climatic zones, recent studies revealed variations in the prevalence of both ascarid and strongylid nematodes, faecal egg shedding, and strongylid species composition (Abbas et al., 2023a, 2024a). Seasonal variation in FECs was particularly pronounced, with climatic influences contributing not only to differences in egg shedding rates but also in the relative abundance of cyathostomin species. For instance, *Cya. catinatum* is highly abundant and consistently reported as one of the top three most prevalent cyathostomins in Europe and North America but was found to be less abundant in recent studies from Australia and Thailand, where it did not rank among the top three species (Abbas et al., 2023a, 2024a; Hamad et al., 2024a). Similarly, a systematic review by Bellaw and Nielsen (2020) reported that *Cys. longibursatus* comprised only 7 % of cyathostomin communities in Eastern Europe, compared to 33 % in North America. These findings highlight substantial regional variation in cyathostomin species composition, suggesting that certain species may be better adapted to specific climatic conditions. Although this emerging evidence underscores the relevance of climate in shaping parasite communities, more work is needed to understand the impact of climate on parasite transmission patterns, species diversity and implications for equine health. In addition to ascarid and strongylid nematodes, it would be beneficial to understand the seasonal and climatic impacts on *A. perfoliata* transmission.

Recent national guidelines for equine internal parasite control (Beasley et al., 2025) strongly advocate for targeted treatment approaches such as TST to reduce anthelmintic use and delay the development of AR. These strategies recommend treating only individual horses that exceed a FEC threshold or display clinical signs of parasitism, thereby preserving refugia - a population of worms not exposed to anthelmintics and is believed to dilute resistant genotypes and slow AR development (Hodgkinson et al., 2019). However, in some scenarios, the effectiveness of TST can vary depending on climatic, environmental and management conditions and may not be feasible, as recently shown in tropical conditions in Cuba (Gómez-Cabrera et al., 2021). In line with this, the global equine parasite control guidelines (Nielsen et al., 2025a) underscore that while the broad international consensus promotes surveillance-based approaches, region-specific environmental factors, particularly climatic conditions significantly influence parasite transmission dynamics and must inform local strategies. Australia presents unique challenges in this regard, with its diverse climatic zones ranging from temperate to tropical conditions and the absence of a defined grazing season. Year-round grazing could facilitate continuous pasture contamination and persistent high larval burdens, which may necessitate more frequent treatment interventions, rendering TST approaches impractical or insufficient in certain regions. Therefore, routine surveillance and context-specific treatment regimens are likely to be essential in tropical and subtropical environments to maintain effective parasite control while minimising the risk of resistance development. Moreover, integrated pasture and farm management practices play a vital role in sustainable parasite control by interrupting the parasite life cycle and reducing pasture contamination (Sallé et al., 2017). Previously, it has been demonstrated that twice-weekly faecal removal can significantly reduce the larval burdens on the pasture, and resting pastures for at least one year is also effective in lowering reinfection pressure (Osterman-Lind et al., 2022). Additionally, mixed grazing with other livestock (e.g., cattle) has been shown to significantly reduce strongyle egg shedding in horses (Forteau et al., 2020). Despite these promising findings, more intensive research is needed to better understand these dynamics across different climatic zones and management systems within Australia. Such research will also be critical in evaluating the uptake and effectiveness of the Australian parasite control guidelines in real-world industry settings.

Another important area for future research could be the identification of immunogenic parasite proteins capable of eliciting long term protective immune responses. This remains a challenging objective due to the complex multicellular nature of helminths and their sophisticated immune evasion strategies. Nevertheless, parasitic helminths excrete or secrete (ES) various molecules into their mammalian hosts. In some host-parasite systems, ES products can induce host-protective immune responses and represent a valuable source of vaccine candidate antigens (Lightowlers and Rickard, 1988). Recent studies have identified promising ES molecules, such as microribonucleic acids (miRNAs), extracellular vesicles (Zakeri et al., 2021) and immunomodulatory proteins, that have been found to be associated with antigen-antibody interactions (Hansen et al., 2019). For instance, Burk et al. (2014) demonstrated ES products derived from *in vitro* cultured second- and/or third-stage larvae of *Parascaris* spp. were reactive with sera from naturally infected foals. While these findings are preliminary, they can potentially lead to the development of serological diagnostic tests and vaccines targeting *Parascaris* infections in horses.

In addition to regular parasite surveillance, monitoring of drug efficacy and improved husbandry and management practices, establishing a national equine parasite database linked to various stakeholders (e.g., industry bodies, veterinary practices, research institutions, diagnostic laboratories) could enable real-time mapping of species diversity, resistance prevalence and emerging trends. Integrating molecular diagnostic tools into this network would further enhance the ability to track resistance-associated alleles and identify emerging parasite species. Investment in such collaborative infrastructure is crucial not only for safeguarding equine health and preserving anthelmintic efficacy but also for supporting global efforts to monitor and mitigate AR.

## CRediT authorship contribution statement

**Ghazanfar Abbas:** Writing – review & editing, Writing – original draft, Formal analysis, Conceptualization. **Martin K. Nielsen:** Writing – review & editing, Writing – original draft, Data curation, Conceptualization. **Charles E-Hage:** Writing – review & editing, Resources. **Abdul Ghafar:** Writing – review & editing, Resources, Formal analysis. **Ian Beveridge:** Writing – review & editing, Supervision. **Jenni Bauquier:** Writing – review & editing, Investigation. **Anne Beasley:** Writing – review & editing, Supervision. **Edwina J.A. Wilkes:** Writing – review & editing, Data curation. **Peter Carrigan:** Writing – review & editing, Data curation. **Lucy Cudmore:** Writing – review & editing, Resources. **Caroline Jacobson:** Writing – review & editing, Supervision. **Kristopher J. Hughes:** Writing – review & editing, Conceptualization. **Abdul Jabbar:** Writing – review & editing, Writing – original draft, Visualization, Supervision, Formal analysis, Conceptualization.

## Declaration of competing interests

The authors of this manuscript are members of the Australian Equine Parasitology Advisory Panel (AEPAP), including Abdul Jabbar, Ghazanfar Abbas, Jenni Bauquier, Charles El-Hage, Abdul Ghafar and Ian Beveridge (The University of Melbourne), Anne Beasley (University of Queensland), Kristopher Hughes (Charles Sturt University), Caroline Jacobson (Murdoch University), Edwina Wilkes (Racing Victoria), and Peter Carrigan and Lucy Cudmore (Scone Equine Hospital). Boehringer Ingelheim supported the panel.

The authors declare that they have no known competing financial interests or personal relationships that could have appeared to influence the work or views reported in this paper.
